# Prize Essay on the Use and Abuse of Alcoholic Liquors, in Health and Disease

**Published:** 1854-07

**Authors:** 


For Sale by MILLER, ORTON & MULLIGAN. H.

---

ART. VIII.—*Prize Essay on the Use and Abuse of Alcoholic Liquors, in Health and Disease.* By WM. B. CARPENTER, M. D., F. R. S., etc., etc. With a preface by D. F. CONDIE, M. D. Philadelphia: Blanchard & Lea. 1851.

Messrs. Blanchard and Lea have issued this much talked of monograph, in a cheap, but neat and substantial form. It is quite the best temperance essay we have ever read. Purely scientific in its style, and moderate in argument, showing no disposition to distort facts to make out a case; it still assumes a ground as high as any tee-totaller could ask, and, which is better, maintains it by irrefutable reasoning.

We are glad to notice that Dr. Carpenter opposes that fanatical devotion to temperance, which would exclude alcohol from our materia medica. He assigns to it a high place among remedies; and does so on the philosophical ground, that it is the best and most convenient stimulus we have in depressed states, particularly of the ganglionic system.

We recommend this book to the physician, not only as a good argument on the right side of a noble cause, but, also, because it affords a clear insight into the mode of action of alcohol upon the system. We were especially struck with the *resume* given of its relations to oleaginous and albuminous food, and, to the treatment of phthisis pulmonalis, and albuminoid diseases.

